# Supplementary material for: Improving microbial fitness in the mammalian gut by in vivo temporal functional metagenomics
Source: Mol Syst Biol. 2015 Mar 11;11(3):788. doi: 10.15252/msb.20145866 (PMC4380924; doi:10.15252/msb.20145866)
Supplement: Supplementary file 1 — Supplementary Table S1 [file MSB-11-788-s001.docx]

# Table S1. Bt genes significantly enriched or depleted at Day 6 or 7 *in vitro*.

Statistically significant genes (q < 0.05) enriched (blue) or depleted (red) at Day 6 or 7 relative to Day 0 in the *in vitro* passaging experiment are listed for the anaerobic mouse chow (MC) and aerobic Luria broth (LB) conditions.

| **Gene** | **Gene product** | **log2(fold change)** | **q value** |
| --- | --- | --- | --- |
| **Enrichment at Day 6 in anaerobic MC passaging** | | | |
| BT_0370 | galactokinase | 3.51 | 8.30E-05 |
| BT_0372 | aldose 1-epimerase | 3.21 | 8.30E-05 |
| BT_0371 | glucose/galactose transporter | 3.59 | 1.53E-04 |
| BT_0478 | hypothetical protein | 3.59 | 3.70E-04 |
| BT_0369 | endo-1,4-beta-xylanase D | 2.51 | 2.63E-03 |
|  |  |  |  |
| **Enrichment at Day 7 in aerobic LB passaging** | | | |
| BT_1750 | glycine betaine/L-proline transport system permease | 9.18 | 0.00E+00 |
| BT_2055 | biopolymer transport protein | 3.64 | 3.03E-05 |
| BT_4358 | hypothetical protein | 2.84 | 6.31E-03 |
| BT_1922 | N-acetylmuramoyl-L-alanine amidase | 2.62 | 7.24E-03 |
| BT_0659 | hypothetical protein | 2.59 | 7.24E-03 |
| BT_4333 | hypothetical protein | 2.57 | 7.84E-03 |
| BT_2054 | hypothetical protein | 2.90 | 8.97E-03 |
| BT_0757 | beta-galactosidase | 3.00 | 1.44E-02 |
| BT_0660 | hypothetical protein | 2.36 | 1.48E-02 |
| BT_2732 | hypothetical protein | 2.48 | 1.48E-02 |
| BT_2843 | integrase | 3.34 | 1.51E-02 |
| BT_3927 | hypothetical protein | 2.43 | 1.56E-02 |
| BT_3612 | FKBP-type peptidylprolyl isomerase | 2.23 | 2.36E-02 |
| BT_3821 | 5,10-methylenetetrahydrofolate reductase | 2.31 | 2.36E-02 |
| BT_0865 | chitobiase | 2.39 | 2.36E-02 |
| BT_0973 | hypothetical protein | 2.21 | 2.36E-02 |
| BT_0676 | N-acetylglucosamine-6-phosphate deacetylase | 2.17 | 2.59E-02 |
| BT_2408 | LuxR family transcriptional regulator | 2.22 | 2.59E-02 |
| BT_1038 | hypothetical protein | 2.24 | 2.59E-02 |
| BT_3985 | hypothetical protein | 2.16 | 2.59E-02 |
| BT_2917 | hypothetical protein | 2.16 | 2.80E-02 |
| BT_0972 | oxidoreductase | 2.13 | 3.17E-02 |
| BT_1923 | O-acetylhomoserine (thiol)-lyase | 2.43 | 3.35E-02 |
| BT_1006 | nitroreductase | 2.10 | 3.64E-02 |
| BT_1004 | hypothetical protein | 2.11 | 3.94E-02 |
| BT_4544 | transposase | 1.99 | 4.64E-02 |
| BT_2379 | hypothetical protein | 5.81 | 4.64E-02 |
| BT_0974 | hypothetical protein | 2.08 | 4.64E-02 |
| BT_0011 | hypothetical protein | 2.00 | 4.64E-02 |
| BT_0510 | heme biosynthesis protein | 2.22 | 4.64E-02 |
|  |  |  |  |
| **Depletion at Day 6 in anaerobic MC passaging** | | | |
| BT_1771 | cell surface protein | -3.25 | 3.70E-04 |
| BT_4572 | phosphopyruvate hydratase (enolase) | -3.48 | 1.32E-03 |
| BT_2959 | hypothetical protein | -2.97 | 5.18E-03 |
| BT_3089 | hypothetical protein | -2.73 | 7.92E-03 |
| BT_3528 | hypothetical protein | -2.95 | 1.69E-02 |
| BT_2051 | hypothetical protein | -4.21 | 1.69E-02 |
| BT_3577 | hypothetical protein | -2.16 | 2.01E-02 |
|  | | | |
| **Depletion at Day 7 in aerobic LB passaging** | | | |
| BT_4572 | phosphopyruvate hydratase (enolase) | -3.17 | 2.78E-03 |
| BT_1538 | hemagglutinin | -3.46 | 7.24E-03 |
| BT_3395 | acetylglutamate kinase | -2.42 | 1.48E-02 |
| BT_1817 | RNA polymerase ECF-type sigma factor | -2.19 | 2.36E-02 |
| BT_1818 | hypothetical protein | -2.54 | 2.36E-02 |
| BT_2961 | hypothetical protein | -2.32 | 2.59E-02 |
| BT_4571 | RNA polymerase ECF-type sigma factor | -3.91 | 3.31E-02 |
| BT_2959 | hypothetical protein | -2.18 | 3.45E-02 |
