# Supplementary material for: Improving microbial fitness in the mammalian gut by in vivo temporal functional metagenomics
Source: Mol Syst Biol. 2015 Mar 11;11(3):788. doi: 10.15252/msb.20145866 (PMC4380924; doi:10.15252/msb.20145866)
Supplement: Supplementary file 2 — Supplementary Table S2 [file MSB-11-788-s002.docx]

# Table S2. Primers used in the study.

| **Name** | **Sequence (5’ -> 3’)** |
| --- | --- |
| A_L | AGGACGCACTGACCGAATT |
| A_R | TTTATTTGATGCCTCTAGCACGC |
| ver2_f | TTTACTTTGCAGGGCTTCCC |
| ver2_r | ACTGAGCCTTTCGTTTTATTTGATG |
| galK16_chk_f | CCTGCCACTCACACCATTCAG |
| galK16_chk_r | TGGGCGCATCGAGGGA |
| GMV_amp_f | AACAAGCTTGATATCGAATTCCTGC |
| GMV_amp_r | GACGGTACCTTTCTCCTCTTTAATGA |
