# Supplementary material for: Improving microbial fitness in the mammalian gut by in vivo temporal functional metagenomics
Source: Mol Syst Biol. 2015 Mar 11;11(3):788. doi: 10.15252/msb.20145866 (PMC4380924; doi:10.15252/msb.20145866)
Supplement: Supplementary file 3 — Supplementary Table S3 [file MSB-11-788-s003.docx]

# Table S3. Summary of sequencing metrics for *in vitro* experiments.

Paired-end reads of 250 nt length were generated on the MiSeq instrument.

| **Media condition** | **Timepoint (day)** | **Paired raw reads** | **Paired trimmed reads** |
| --- | --- | --- | --- |
| input library | 0 | 5675769 | 5671724 |
| LB aerobic | 7 | 8226863 | 8222571 |
| MC anaerobic | 6 | 6672316 | 6668006 |
