# Supplementary material for: Improving microbial fitness in the mammalian gut by in vivo temporal functional metagenomics
Source: Mol Syst Biol. 2015 Mar 11;11(3):788. doi: 10.15252/msb.20145866 (PMC4380924; doi:10.15252/msb.20145866)
Supplement: Supplementary file 4 — Supplementary Table S4 [file MSB-11-788-s004.docx]

# Table S4. Summary of sequencing metrics for *in vivo* experiments.

Of the 56 samples sequenced, two were the input library, 44 were from four mice with 11 time-point stool collections, and 10 were from one mouse with a 10 time-point collection. Paired-end reads of 101 nt length were generated on the HiSeq instrument.

| **Mouse** | **Timepoint (day)** | **Paired raw reads** | **Paired trimmed reads** |
| --- | --- | --- | --- |
| 1 | 1.5 | 6863895 | 6859853 |
| 1 | 1.75 | 7759215 | 7751437 |
| 1 | 2.5 | 7365279 | 7362905 |
| 1 | 3 | 5818449 | 5818061 |
| 1 | 4 | 5531304 | 5530269 |
| 1 | 7 | 5662825 | 5662593 |
| 1 | 10 | 6698408 | 6697569 |
| 1 | 14 | 4723483 | 4723238 |
| 1 | 21 | 6687984 | 6687697 |
| 1 | 28 | 5839481 | 5839205 |
| 2 | 0.5 | 7228693 | 7225763 |
| 2 | 1.5 | 8918192 | 8916633 |
| 2 | 1.75 | 5888569 | 5887816 |
| 2 | 2.5 | 8785555 | 8784833 |
| 2 | 3 | 4342612 | 4342118 |
| 2 | 4 | 6837676 | 6835603 |
| 2 | 7 | 3967877 | 3967384 |
| 2 | 10 | 8601605 | 8601131 |
| 2 | 14 | 5849784 | 5849571 |
| 2 | 21 | 4556363 | 4556235 |
| 2 | 28 | 7655382 | 7655090 |
| 3 | 0.5 | 5010698 | 5005943 |
| 3 | 1.5 | 6155822 | 6155291 |
| 3 | 1.75 | 12620630 | 12619054 |
| 3 | 2.5 | 4684094 | 4683664 |
| 3 | 3 | 6101414 | 6100076 |
| 3 | 4 | 5942854 | 5942213 |
| 3 | 7 | 4172374 | 4171934 |
| 3 | 10 | 4801895 | 4801689 |
| 3 | 14 | 2164395 | 4812691 |
| 3 | 21 | 4812865 | 7188598 |
| 3 | 28 | 7188952 | 2164338 |
| 4 | 0.5 | 4533216 | 4526163 |
| 4 | 1.5 | 4334677 | 4331837 |
| 4 | 1.75 | 7468959 | 7466817 |
| 4 | 2.5 | 4985966 | 4984355 |
| 4 | 3 | 5103796 | 5102782 |
| 4 | 4 | 9074708 | 9073032 |
| 4 | 7 | 6749347 | 6748350 |
| 4 | 10 | 5542079 | 5541171 |
| 4 | 14 | 4411316 | 4410997 |
| 4 | 21 | 7574141 | 7573540 |
| 4 | 28 | 6262455 | 6262057 |
| 5 | 0.5 | 7060331 | 7051476 |
| 5 | 1.5 | 5588814 | 5583481 |
| 5 | 1.75 | 4776273 | 4771776 |
| 5 | 2.5 | 6594126 | 6590706 |
| 5 | 3 | 5617337 | 5616357 |
| 5 | 4 | 6090548 | 6089696 |
| 5 | 7 | 8025711 | 8024678 |
| 5 | 10 | 4246859 | 4246609 |
| 5 | 14 | 5401171 | 5400825 |
| 5 | 21 | 4771095 | 4770757 |
| 5 | 28 | 3923280 | 3922897 |
| input library | replicate 1 | 7305414 | 7303389 |
| input library | replicate 2 | 3852825 | 3849575 |
