# Supplementary material for: Improving microbial fitness in the mammalian gut by in vivo temporal functional metagenomics
Source: Mol Syst Biol. 2015 Mar 11;11(3):788. doi: 10.15252/msb.20145866 (PMC4380924; doi:10.15252/msb.20145866)
Supplement: Supplementary file 5 — Supplementary Table S5 [file MSB-11-788-s005.docx]

# Table S5. Summary of metrics for whole genome sequencing of *E. coli* strains.

Paired-end reads of 300 nt length were generated on the MiSeq instrument.

| **Sample** | **Paired raw reads** |
| --- | --- |
| NEB Turbo control | 357506 |
| Day 7 Mouse 1 clone 3 | 376194 |
| Day 7 Mouse 1 clone 1 | 785257 |
| Day 7 Mouse 2 clone 5 | 1291948 |
| Day 7 Mouse 3 clone 1 | 1257935 |
| Day 7 Mouse 4 clone 4 | 1133157 |
| Day 7 Mouse 5 clone 2 | 1049238 |
| Day 7 Mouse 5 clone 4 | 170610 |
| Day 28 Mouse 1 clone 1 | 1112924 |
| Day 28 Mouse 2 clone 1 | 574731 |
| Day 28 Mouse 3 clone 1 | 543707 |
| Day 28 Mouse 4 clone 1 | 618713 |
| Day 28 Mouse 5 clone 1 | 169684 |
| Day 28 Mouse 5 clone 4 | 687173 |
| Day 28 Mouse 7 clone 1 lux control | 1194434 |
| Day 28 Mouse 10 clone 2 lux control | 931238 |
