# Supplementary figures and images for: Improving microbial fitness in the mammalian gut by in vivo temporal functional metagenomics
Source: Mol Syst Biol. 2015 Mar 11;11(3):788. doi: 10.15252/msb.20145866 (PMC4380924; doi:10.15252/msb.20145866)

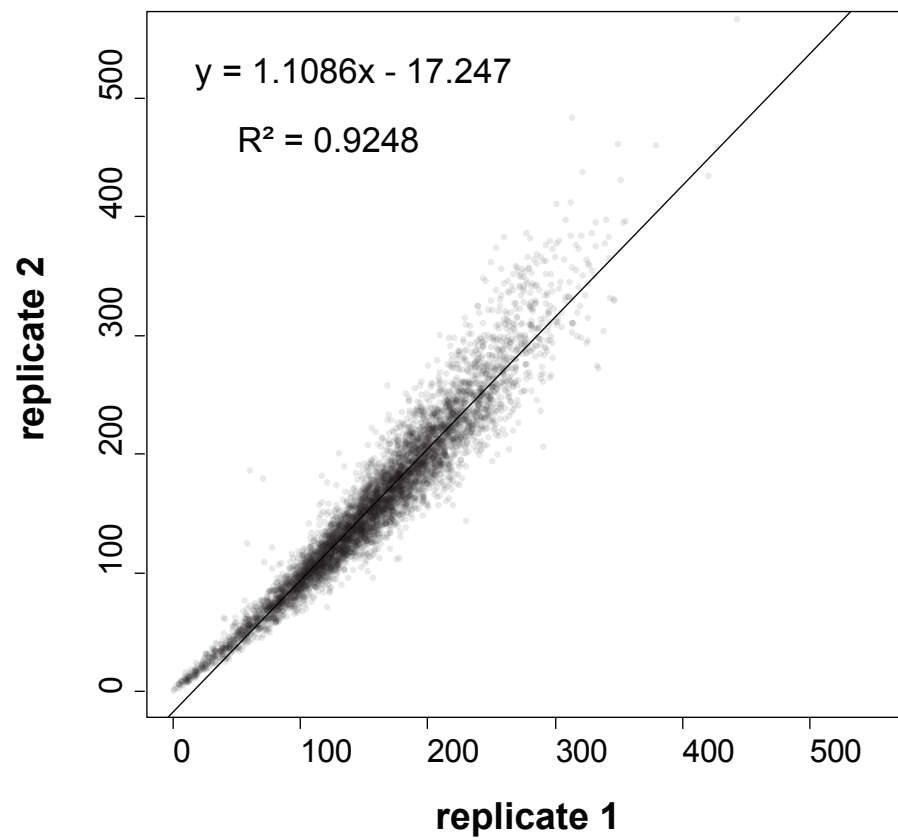

Supplement: Supplementary file 6 — Supplementary Figure S1 [file MSB-11-788-s006.pdf]

**A**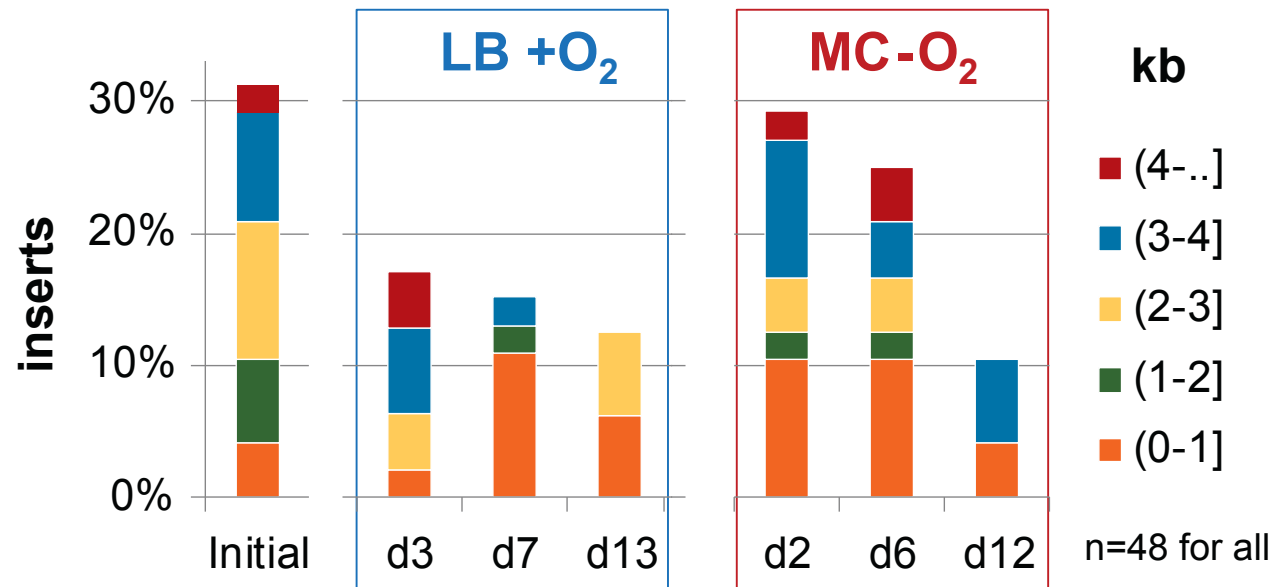**B**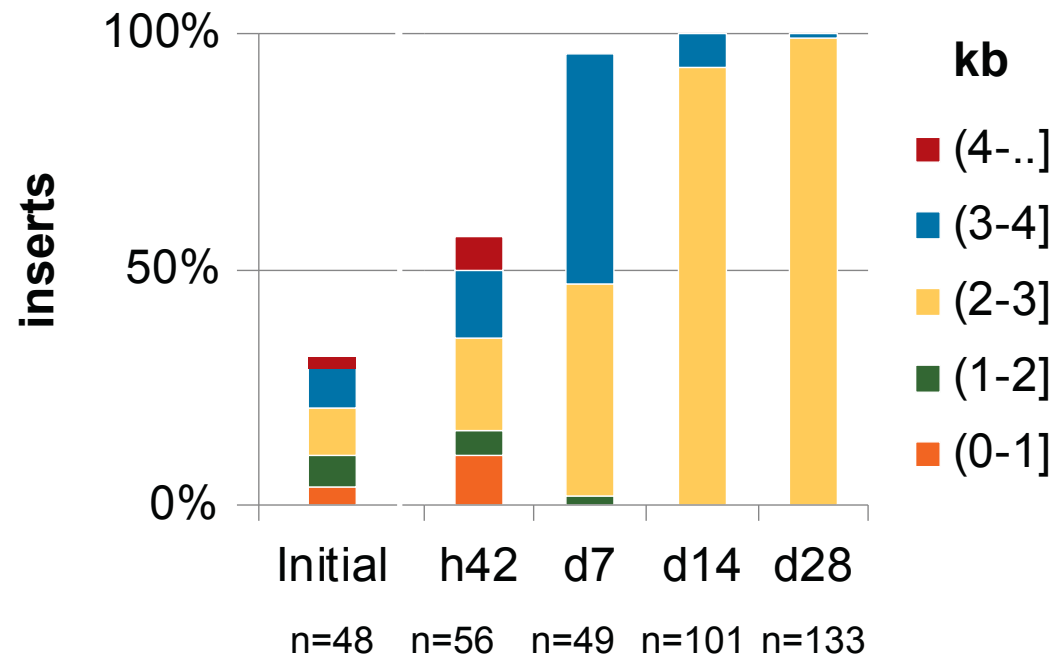

Supplement: Supplementary file 7 — Supplementary Figure S2 [file MSB-11-788-s007.pdf]

# % of mapped bases

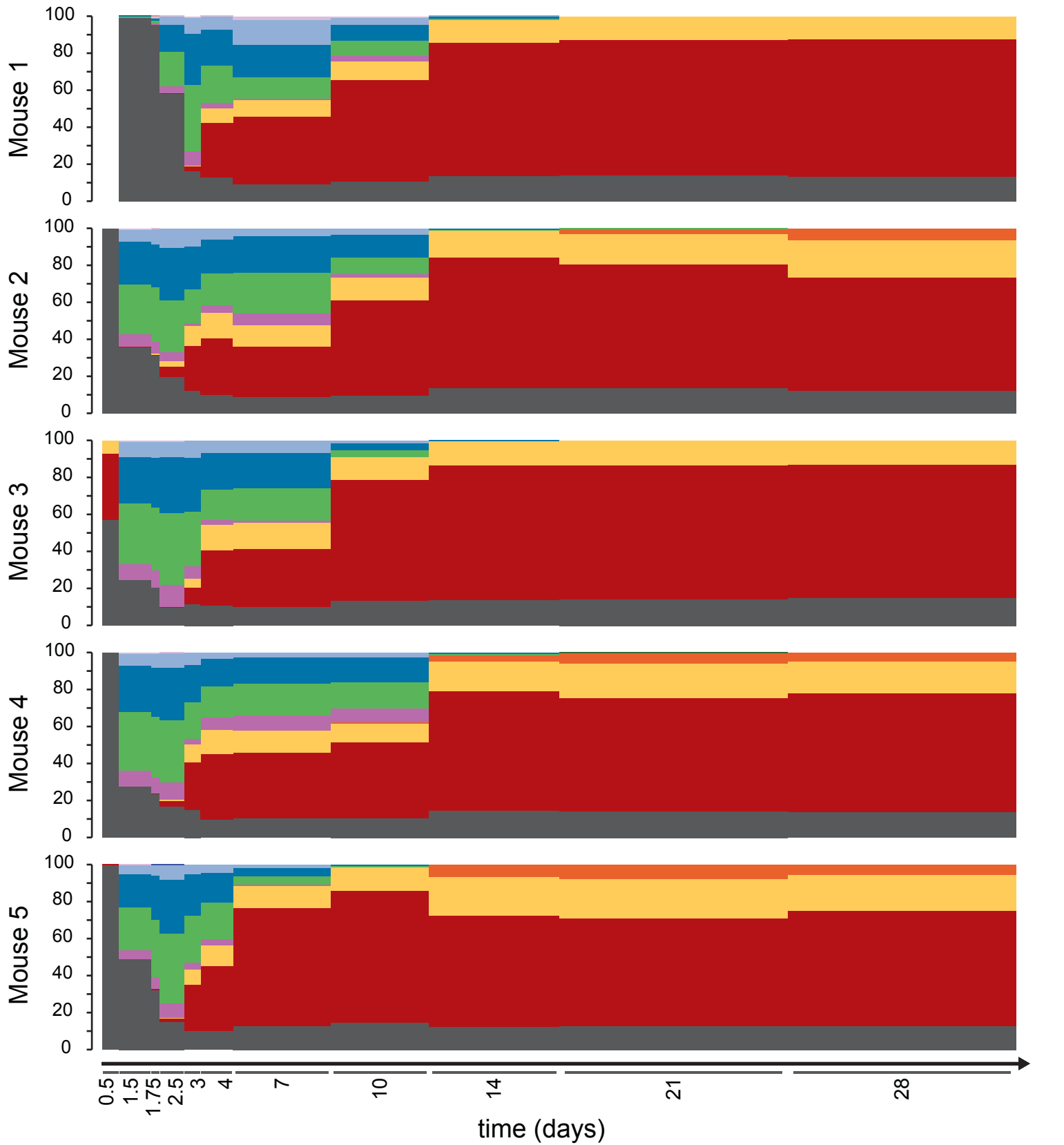

Supplement: Supplementary file 8 — Supplementary Figure S3 [file MSB-11-788-s008.pdf]

**A**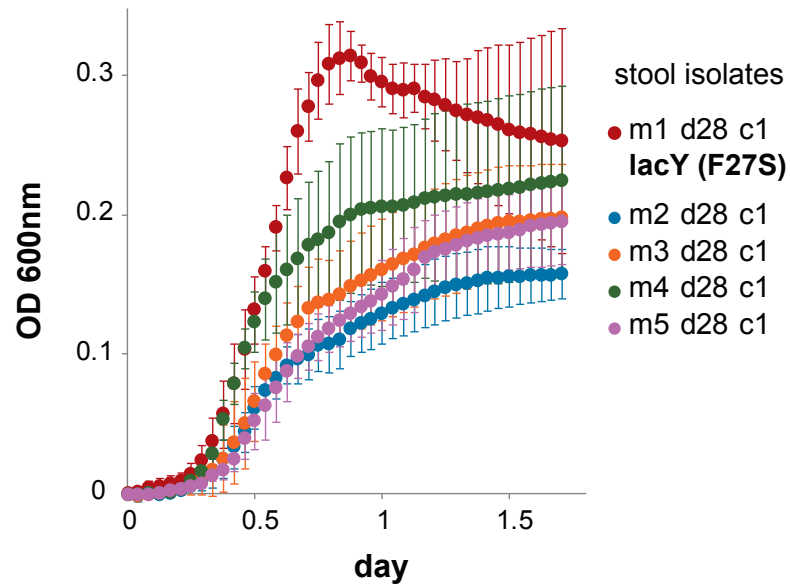**B**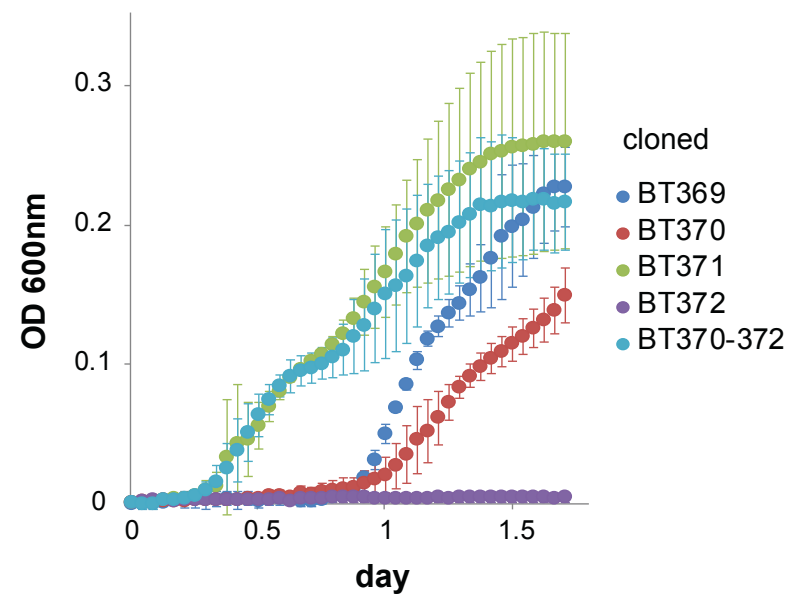**C**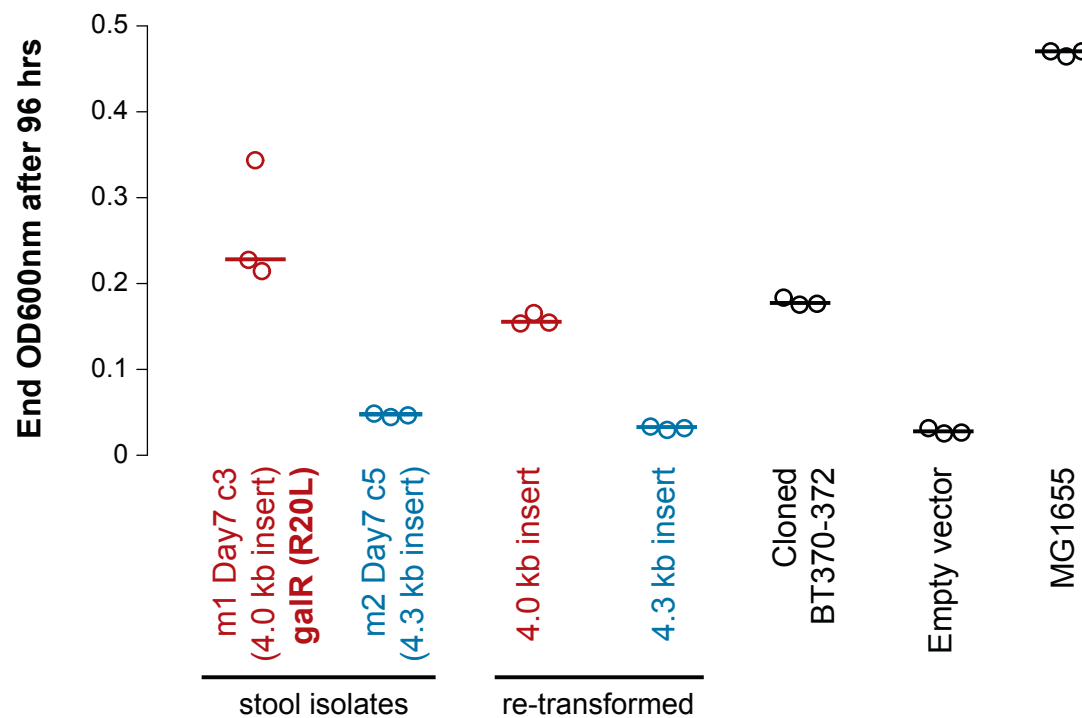

Supplement: Supplementary file 10 — Supplementary Figure S5 [file MSB-11-788-s010.pdf]
