# Supplementary material for: Improving microbial fitness in the mammalian gut by in vivo temporal functional metagenomics
Source: Mol Syst Biol. 2015 Mar 11;11(3):788. doi: 10.15252/msb.20145866 (PMC4380924; doi:10.15252/msb.20145866)
Supplement: Supplementary file 9 — Supplementary Figure S4 [file MSB-11-788-s009.pdf]

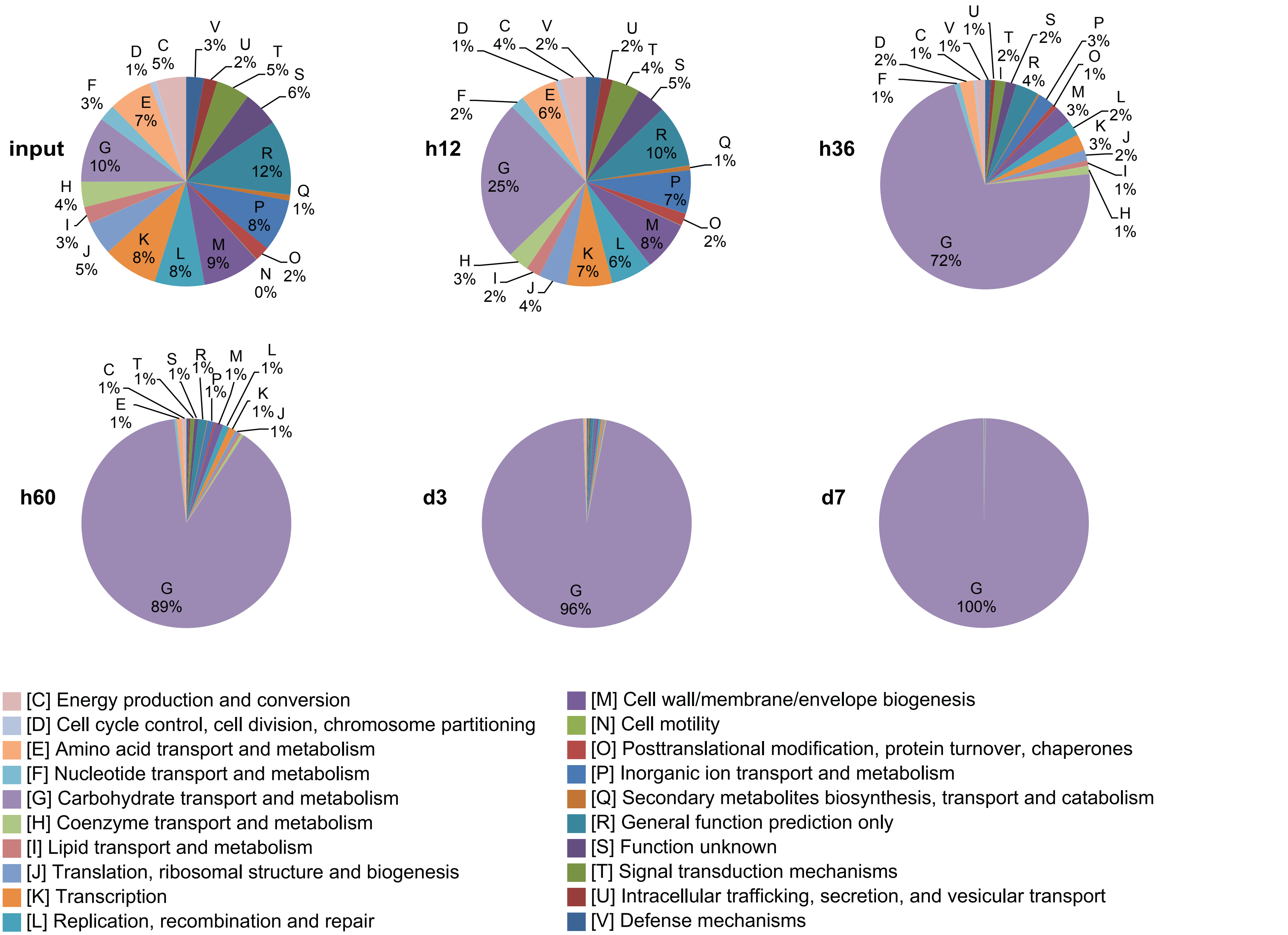

input
C 5%
D 1%
F 3%
E 7%
G 10%
H 4%
I 3%
J 5%
K 8%
L 8%
M 9%
N 0%
O 2%
P 8%
Q 1%
R 12%
S 6%
T 5%
U 2%
V 3%
h12
D 1%
C 4%
V 2%
U 2%
T 4%
S 5%
F 2%
E 6%
R 10%
Q 1%
G 25%
P 7%
O 2%
M 8%
L 6%
K 7%
J 4%
I 2%
H 3%
h36
D 2%
C 1%
V 1%
U 1%
T 2%
S 2%
P 3%
F 1%
I
R 4%
O 1%
M 3%
L 2%
K 3%
J 2%
I 1%
H 1%
G 72%
h60
C 1%
T 1%
S 1%
R 1%
M 1%
L 1%
E 1%
P 1%
K 1%
J 1%
G 89%
d3
G 96%
d7
G 100%
[C] Energy production and conversion
[D] Cell cycle control, cell division, chromosome partitioning
[E] Amino acid transport and metabolism
[F] Nucleotide transport and metabolism
[G] Carbohydrate transport and metabolism
[H] Coenzyme transport and metabolism
[I] Lipid transport and metabolism
[J] Translation, ribosomal structure and biogenesis
[K] Transcription
[L] Replication, recombination and repair
[M] Cell wall/membrane/envelope biogenesis
[N] Cell motility
[O] Posttranslational modification, protein turnover, chaperones
[P] Inorganic ion transport and metabolism
[Q] Secondary metabolites biosynthesis, transport and catabolism
[R] General function prediction only
[S] Function unknown
[T] Signal transduction mechanisms
[U] Intracellular trafficking, secretion, and vesicular transport
[V] Defense mechanisms
