# Supplementary material for: Improving microbial fitness in the mammalian gut by in vivo temporal functional metagenomics
Source: Mol Syst Biol. 2015 Mar 11;11(3):788. doi: 10.15252/msb.20145866 (PMC4380924; doi:10.15252/msb.20145866)
Supplement: Supplementary file 11 — Supplementary Figure S6 [file MSB-11-788-s011.pdf]

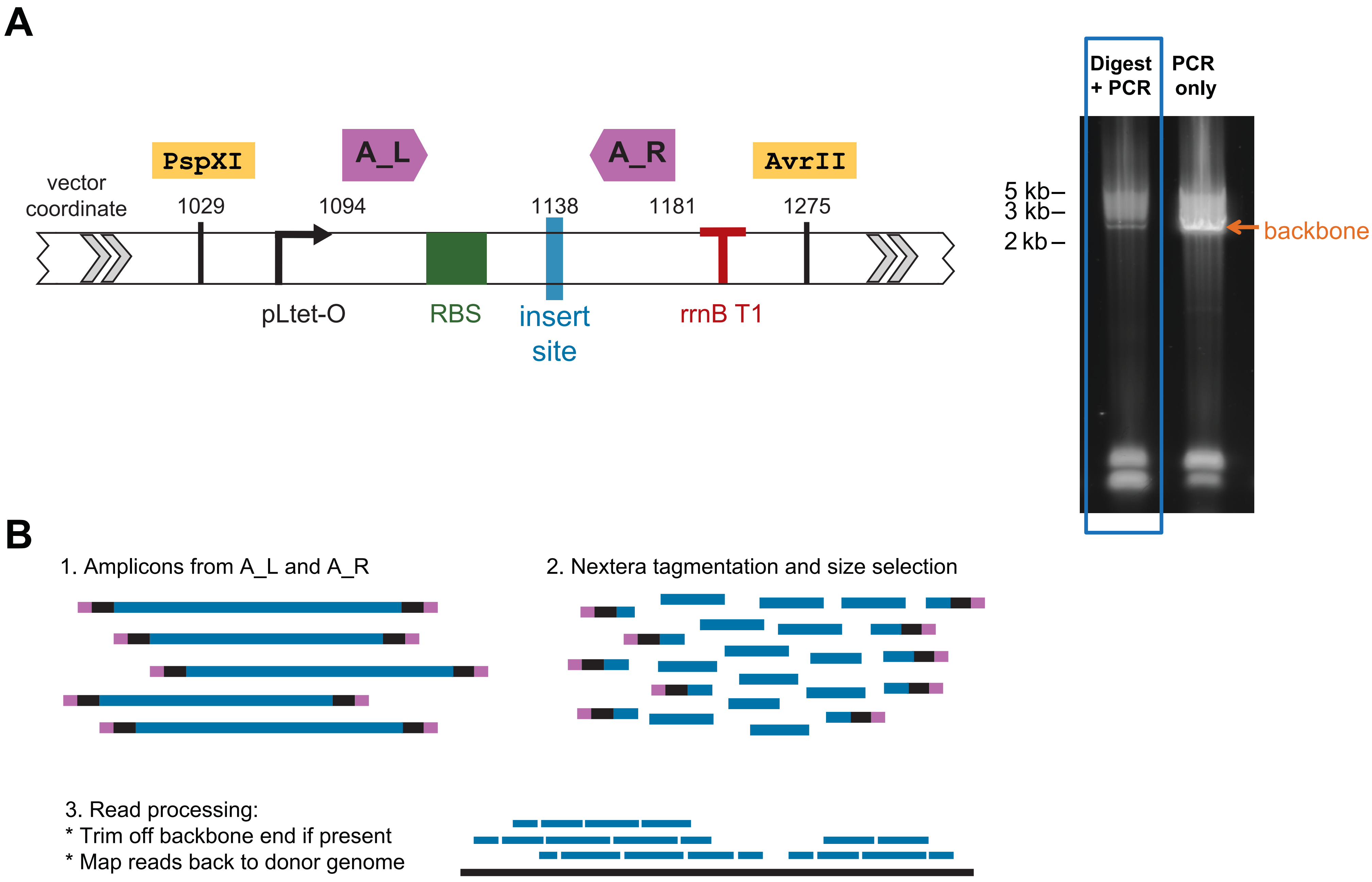

A
PspXI
A_L
A_R
AvrII
vector coordinate
1029
1094
1138
1181
1275
pLtet-O
RBS
insert site
rrnB T1
Digest + PCR
PCR only
5 kb
3 kb
2 kb
backbone
B
1. Amplicons from A_L and A_R
2. Nextera tagmentation and size selection
3. Read processing:
* Trim off backbone end if present
* Map reads back to donor genome
