# Supplementary material for: Improving microbial fitness in the mammalian gut by in vivo temporal functional metagenomics
Source: Mol Syst Biol. 2015 Mar 11;11(3):788. doi: 10.15252/msb.20145866 (PMC4380924; doi:10.15252/msb.20145866)
Supplement: Supplementary file 14 — Supplementary Figure Legends [file MSB-11-788-s014.docx]

Supplementary Figure legends

Figure S1. Technical reproducibility of library sequencing protocol. The input library was prepared in duplicate for deep sequencing using our double digestion and PCR strategy. The coverage for each gene in replicate 1 is plotted against that of replicate 2.

Figure S2. Distribution of inserts in the initial library and at various time points in the **(A)** *in vitro* and **(B)** *in vivo* experiments. Multiple colonies were picked from each mouse and the total insert sizes were tabulated for each time point.

Figure S3. Distribution of mapped bases to each Bt gene by mouse. For each mouse and time point, ~10^9^ sequenced bases were mapped to the *B. thetaiotaomicron* genome. Of those mapped bases, the percentage mapping to each gene is shown. Genes with < 0.2% are grouped together (dark gray bars). Specific genes >= 0.2% that were present in clones in one mouse but not in the others are indicated in smaller font and colored differently.

# Figure S4. COG functional categories of bases mapped to the entire Bt genome averaged across the five mice.

Figure S5. Growth characterization of clones with genomic SNVs. Growth curves over 42 hours at 37^o^C in M9 with 0.2% galactose and carbenicillin of **(A)** mouse-isolated clones from Day 28 and **(B)** BT_0369, BT_0370, BT_0371, BT_0372, and BT_0370-BT_0372 cloned into the starting recipient *E. coli* strain. The mean of four replicates is plotted in filled circles; error bars represent the standard deviation. **(C)** Endpoint optical density after 96 hours of growth. Two mouse-isolated strains with the BT_0370 insert were compared to isogenic strains transformed with those plasmids (4.0 or 4.3 kb insert). The strain with the galR SNV is shown in red. Lines represent the mean.

Figure S6. Double digestion and PCR protocol for sequencing. **(A)** We used restriction sites PspXI and AvrII that flanked the insert site on the backbone vector prior to PCR-amplification of the insert with primers A_L and A_R. These two enzymes had a minimal number of restriction sites (29 for PspXI and 62 for AvrII) in the *B. thetaiotaomicron* genome. The gel shows the result of library PCR with or without double digestion. Double digestion appears to eliminate the dominating band corresponding to the backbone vector. **(B)** PCR amplicons were prepared for sequencing by the Nextera kit and size selection. After trimming off any backbone sequence, which would be present on end fragments, we mapped the reads back to the *B. thetaiotaomicron* genome.
