# Supplementary material for: Improving microbial fitness in the mammalian gut by in vivo temporal functional metagenomics
Source: Mol Syst Biol. 2015 Mar 11;11(3):788. doi: 10.15252/msb.20145866 (PMC4380924; doi:10.15252/msb.20145866)
Supplement: Supplementary file 15 — Supplementary Materials and Methods [file MSB-11-788-s015.docx]

**Supplementary Materials and Methods**

**Table of Contents**

[Media recipe for *Bacteroides thetaiotaomicron* 2](#_Toc400846366)

[Genomic library preparation 3](#_Toc400846367)

[Library PCR protocol 7](#_Toc400846368)

# Media recipe for *Bacteroides thetaiotaomicron*

For 1 Liter:

22.2 g BHI (3/5 X)

22 g MRS (2/5 X)

20 g Trypicase peptone (2/3 X)

10 g Tryptose

3 g Soytone

 1 g L-cysteine

10 mL Hemin solution

1 mL Resazurin solution

0.2 mL Vitamin K1 solution

1 g Soluble Starch

** Add after autoclaving **

** 20 mL NaHCO3 solution

** 10 mL sugar solution (1 g fructose, 1 g cellobiose, 3.42 g maltose)

*Solution formulas:*

Vitamin K1 solution:

15 uL of stock into 3 mL 100% ethanol

Solution good for one month.

Store in 4C, protected from light.

Hemin solution:

For 200 ml: Dissolve 100 mg of hemin in 2 ml of 1 M NaOH and then bring up volume to 200 ml with dH2O. Store in an amber bottle up to 1 year at 4◦C.

NaHCO3, 10% solution:

Dissolve 50 g into 500 ml H2O, filter sterilize.

Resazurin, 0.1% solution

Dissolve 0.1 g of resazurin (Sigma Chemical) in 100 ml distilled water.

# Genomic library preparation

**Vector Preparation**

1. PCR reaction

| Volume | Component |
| --- | --- |
| 10 uL | Nuclease free water |
| 0.75 uL | Forward primer (10uM) (GMV_amp_f) |
| 0.75 uL | Reverse primer (10uM) (GMV_amp_r) |
| 1 uL | Template: expression vector (GMV1c) |
| 12.5 uL | Kapa HiFi hotstart readymix (2x) |
| 25 uL | Total Volume |

| Step | Temp (^o^C) | Time |
| --- | --- | --- |
| 1 | 95 | 3 min |
| 2 | 98 | 20 sec |
| 3 | 64 | 15 sec |
| 4 | 72 | 60 sec |
| 2-4 |  | 20x |
| 5 | 72 | 3 min |
| 6 | 4 | Forever |

1. Purify the reaction using a QiaQuick PCR purification column into 50 uL EB buffer.
2. DpnI digest the elution

| Volume | Component |
| --- | --- |
| 50 uL | Purified PCR product |
| 5.73 | NEB buffer 4 (10x) |
| 1.5 uL | NEB DpnI |

Incubate for 90 minutes at 37C.

1. Run the digestion product on a gel a 1% E-gel EX.
2. Crack open the gel and stab the desired band (between 2.0 and 2.5 kB) with a p20 pipette tip into 50 uL of nuclease free water.
3. Heat the gel stab/water mixture at 90 C for 10 minutes to use as template
4. Prepare a second round PCR reaction using the same conditions as before, except with 26X instead of 20X cycles.
5. PCR purify by Qiaquick column into 50 uL nuclease free H2O
6. Store at -20C until needed

**Insert Preparation**

1. Grow 3 mL of Bacteroides thetaiotaomacron to saturation (Note: may take a few days)
2. Use Qiagen DNeasy Blood and Tissue Kit to extract genomic DNA from 750 uL of culture.
3. Shear DNA to 5kbp by w/ Covaris E210 Machine

| Operating Parameters |  |
| --- | --- |
| Temperature | 19-21 |
| Duty Cycle | 20% |
| Cycles per Burst | 1000 |
| Total Treatment Time | 600 seconds |
| Power mode | Frequency Sweeping |
| Degassing Mode | Continuous |
| Volume | 200 uL |
| Buffer | Tris EDTA, pH 8.0 |
| DNA mass | 2-20 ug |
| Starting material | >50 kb |
| Water level(Fill/Run) | 13 |
| AFA Intensifier | No Intensifier |

1. Prepare a preparatory gel with 0.8% agarose and 100,000x gel green dye (label recommends 10,000 gel green this is to much).
2. Load the whole sample w/ 6x NEB loading buffer.
3. Run the gel at constant voltage between 100 v and 120 v for about 4 hours.
4. Excise 3 kbp – 5kbp range (because of the high DNA concentration sample will likely run a little faster than the ladder.)
5. Use Qiagen Qiaquick Gel Purification Kit to dissolve agarose and purify DNA
6. Assemble End-Repair rxn (Blunt ends and add 5’ phosphates with epicenter End-It DNA End-Repair Kit)
7. Run rxn cycle

| Volume | Component |
| --- | --- |
| 1 uL – 34 uL | Sheared gel purified DNA |
| 5 uL | 10x End-Repair Buffer |
| 5 uL | dNTP mix |
| 5 uL | ATP |
| x uL | Nuclease free water |
| 1 uL | End-Repair enzyme mix |
| 50 uL | Total rxn volume |

| Tempature | Time |
| --- | --- |
| RT | 1 Hour |
| 70 C | 10 Minutes |

1. Clean up rxn with QiaQuick PCR purification column elute into 50 uL nuclease free water
2. Store at -20 C until ready to use
3. Measure concentration of vector and insert, and run out on a gel to confirm sizes.


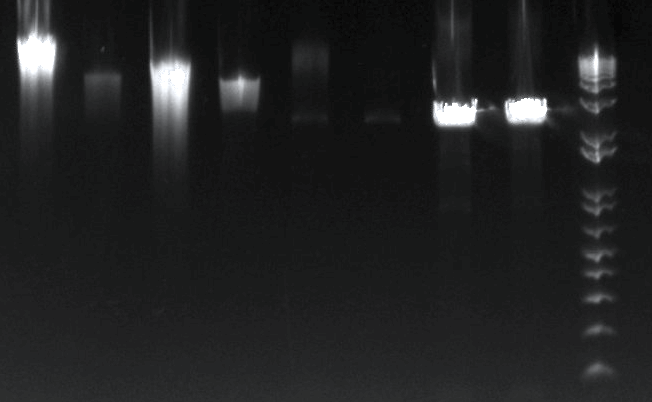
L1: Sheared Bthe (1)

L2: Gel Purified Bthe (1)

L3: Sheared Bthe (2)

L4: Gel Purified Bthe (2)

L5: GMV1c mini prep

L6: GMV1c midi prep

L7: GMV1c linear

L8: GMV1c vector prep (2)

**Ligation and Transformation**

1. Assemble ligations. (run 4x rxns)

| Component | Amount (1x) | Amount (4x) |
| --- | --- | --- |
| Vector | 33.3 ng | 133.2 ng |
| Insert | 166.6 ng | 666.4 ng |
| 10x t4 ligase buffer | 1.5 uL | 6 uL |
| T4 Ligase | 4 uL | 16 uL |
| Nuclease Free H2O | X uL | X uL |
| Total rxn volume | 15 uL | 60 uL |

1. Incubate the rxns at 16 C over night then heat inactivate at 65 C for 10 minutes
2. Clean up and concentrate rxns with QiaQuick PCR purification column by applying 2x ligation rxns per column. Elute each column into 50 uL nuclease free water for a total of 100 uL.
3. Combine the 100 uL elution to 400 uL of NEB turbo electro competent cell prep thawed on ice and mix gently. Be carful not to generate bubbles.
4. Incubate the mixture on ice for 1 minute
5. Transform the whole volume in 10 separate 50 uL Electroporations.
6. Recover the cells in 7.5 mL (750 uL per rxn) of SOC at 37C (shaking) for 1 hour.
7. Plate 1 uL of the recovery on LB carb plates.
8. To select for transformants add the remaining recovery volume to 143 mL LB carb and incubate at 30 C (shaking) to saturation (about 12 hours).
9. Perform a Qiagen plasmid MidiPrep with 50 mL of the culture eluting into 200 uL EB.
10. Store Elution at -20 until needed
11. Flash freeze at least 10 1 mL aliquots in 15% Glycerol
12. Store at -80 C until needed.

**Library Quantitation**

1. Count the colonies on the plate and record the number

Library Size=CFU*7500(uL of recovery)

1. Pick 96 colonies into a 96 well plate of LB carb (200uL/well).
2. Incubate the 96 well plate at 37 C with shaking until saturation.
3. Prepare a your PCR template by diluting each well 100x in nuclease free H2O
4. Assemble 96 well inset detect PCR master Mix

| Component | 1x amount | 100x amount |
| --- | --- | --- |
| Kapa HiFi Hotstart 2x MM | 5 uL | 500 uL |
| GMV1c_ver2_f primer | 0.3 uL | 30 uL |
| GMV1c_ver2_r primer | 0.3 uL | 30 uL |
| Nuclease free H2O | 3.4 uL | 340 uL |

1. Aliquot 9 uL of the PCR master Mix to each well of a 96 well plate
2. Add 1 uL of template from each well of template to its corresponding well on the PCR plate.
3. Seal the PCR plate and run the cycle.

| Step | Temp (^o^C) | Time |
| --- | --- | --- |
| 1 | 95 | 3 min |
| 2 | 98 | 20 sec |
| 3 | 64 | 15 sec |
| 4 | 72 | 2 min |
| 2-4 |  | 26x |
| 5 | 72 | 2 min |
| 6 | 4 | Forever |

1. Dilute the PCR product 2x and run on a 1% gel.

It is likely that some lanes won’t amplify you can re run these samples with a longer extention time to discover the presence of long insert clones (try 4 min)

Empty vectors appear at 250 bp. Larger fragments indicate the presence of an insert.

1. Record the length of each insert (inset length=band size-250).

# Library PCR protocol

**1A. DNA extraction for input library:**

- Start with ~1mL of cultured *E. coli* with the library.
- Use **Qiagen DNeasy Blood & Tissue Kit** (the following is from the Pretreatment Protocol for Gram-Negative Bacteria)
  - Spin at 5000 x g for 10 min at room temperature. Discard supernatant.
  - Resuspend cell pellet in 180uL ATL buffer.
  - Proceed with Step 2 in “Animal Tissue: Spin-Column Protocol”.

**1B. DNA extraction for mouse fecal pellets:**

- (In cold room, transfer stool pellets/homogenates to tubes that fit tabletop centrifuge.)
- Spin down in cold room for 30s at max speed.
- Remove supernatant to another tube (to save as backup) without disturbing pellet.
- Spin down again and remove supernatant.
- Pellet is now ready for DNA extraction using **Qiagen QIAamp DNA Stool Mini Kit**.

1. **DNA digestion:**

- Nanodrop extracted DNA to quantify concentration.
- For the GMV1c vector, double digestion uses two restriction enzymes: **PspXI** and **AvrII**.
- Aim for ~500ng or less DNA in one 50μL reaction. If low concentration of DNA (e.g., 0-10 ng/μL, typical of mouse fecal extractions), use max volume of DNA possible. If high concentration of DNA (e.g., 200 ng/μL, for E. coli library extraction), make up remaining volume with water.

Low [DNA] High [DNA]

10X CutSmart buffer 5 μL 5 μL

DNA 43 μL 2 μL

PspXI 1 μL 1 μL

AvrII 1 μL 1 μL

H_2_O 0 μL 41 μL

- Incubate at 37^o^C for 1 hour.
- Purify digestion reaction with Qiagen column (**QIAquick PCR Purification Kit**). Elute in 40 μL Qiagen EB Buffer.

1. **PCR amplification:**

- Sequences of primers:
  - A_L: AGGACGCACTGACCGAATT
  - A_R: TTTATTTGATGCCTCTAGCACGC
- Reaction volume: 40μL
- Starting material: ~100ng DNA (typically use max volume allowed since already normalized DNA concentrations to max 10 ng/μL from digestion reaction)

Low [DNA] High [DNA]

2X **KAPA HiFi HotStart ReadyMix** 20 μL 20 μL

A_L primer (10μM) 1.2 μL 1.2 μL

A_R primer (10μM) 1.2 μL 1.2 μL

Nucelase-free water 0 μL 10 μL

DNA 17.6 μL 7.6 μL

- Thermocycler protocol:
  - 95^o^C 5 min
  - 98^o^C 20 sec -|
  - 64^o^C 30 sec -| 20 cycles
  - 72^o^C 3 min -|
  - 72^o^C 5 min
  - 8^o^C forever

1. **PCR purification using beads (Agencourt AMPure XP - Beckman Coulter)**

- Follow manufacturer’s protocol. Requires **96-well magnetic magnetic plate**.
- Optimal volumetric ratio: 0.5 beads to 1 sample (20μL beads to 40μL PCR reaction)
- Elute in 40μL Qiagen EB buffer

Optional: run out final sample on a gel for a visual check.
